# Morphological Characterization of the In Vitro Mycorrhizae Formed between Four *Terfezia* Species (*Pezizaceae*) with *Cistus salviifolius* and *Cistus ladanifer*—Towards Desert Truffles Production in Acid Soils

**DOI:** 10.3390/jof7010035

**Published:** 2021-01-09

**Authors:** Rogério Louro, Bruno Natário, Celeste Santos-Silva

**Affiliations:** Biology Department, Macromycology Laboratory, MED—Mediterranean Institute for Agriculture, Environment and Development, University of Évora, 7000-083 Évora, Portugal; rlouro@uevora.pt (R.L.); bafnatario@gmail.com (B.N.)

**Keywords:** desert truffles, *Terfezia* cultivation, *Cistus*, putative plant hosts, mycorrhizae characte-rization, acid soils

## Abstract

*Terfezia* species are obligate symbiotic partners of several xerophytic host plants, mainly belonging to the *Cistaceae*. Yet, their mycorrhizal associations with members of the genus *Cistus* remain poorly characterized and their potential application in desert truffle cultivation remains unexplored. This work provides the first anatomic descriptions of the *mycorrhizae* formed in vitro by four *Terfezia* species (i.e., *T. arenaria*; *T. extremadurensis*; *T. fanfani*, *T. pini*) with *C. ladanifer* and *C. salviifolius*, two of the most widespread and common *Cistus* species in acidic soils. All the tested associations resulted in the formation of ectomycorrhizae with well-developed Hartig net, but with a varying degree of mantle development. Our results also demonstrate that all the experimented *Terfezia-Cistus* combinations expressed high mycorrhization rates. Moreover, the present work shows that *C. salviifolius* and *C. ladanifer* are suitable plant hosts for *Terfezia* species, including some that are, to date, known to be only associated with annual herbs or tree species. This new evidence might aid in broadening the number of situations whereby *Terfezia* spp. can be cultivated in acid soils.

## 1. Introduction

The term “desert truffle” commonly designates the fruitbodies produced by edible hypogeous *Ascomycota* (*Pezizaceae*), which includes several species of the genera *Terfezia*, *Tirmania* and *Picoa*, found in arid and semiarid areas throughout the world [1,2,3]. These fungi establish key mutualistic associations in arid and semiarid ecosystems with the roots of several xerophytic host plants [4], mainly belonging to the *Cistaceae* (e.g., *Helianthemum* spp. and *Cistus* spp.) but also to the *Fagaceae* and *Pinaceae* (i.e., oaks and pines) [2,5,6,7,8].

Many *Terfezia* species are among the most prized desert truffle species. Thus, in the last few decades, numerous research efforts have been made in order to enable their large-scale cultivation [2]. Still, desert truffle cultivation is only now leaving its infancy, and our knowledge on the ecology, physiology, and biochemistry of many *Terfezia* mycorrhizal associations still remains fragmentary [8]. One key issue that remains neglected is the choice of suitable putative hosts. In fact, so far, the only host plants that have been tested in experimental desert truffle cultivation are perennial and annual species of *Helianthemum* “*sensu lato*” and little or no attention has been given to the assessment of new potential hosts for desert truffle cultivation [9]. Within *Cistaceae,* several *Cistus* perennial species are considered putative hosts for *Terfezia* spp. [6,10,11,12] and therefore could be excellent candidates for desert truffle cultivation.

In fact, the genus *Cistus* L. (*Cistaceae*) includes about 20 perennial shrub species, distributed throughout the Mediterranean region and Canary Islands. In the Iberian Peninsula, the genus is represented by 13 species, all belonging to primary successional stages of many forest stands, growing readily in degraded areas or after disturbances such as fire [13,14], rendering their ectomycorrhizal ecology particularly interesting in the context of global warming and increasing desertification, in arid and semiarid areas worldwide.

As a whole, *Terfezia* mycorrhizae are known to display great structural versatility depending on certain factors (i.e., host species, concentration of auxins secreted by the fungi, root sensitivity to those auxins, phosphate concentrations, and drought conditions, [15,16]). Research on the association of *Terfezia* and various *Cistaceae* (mostly perennial and annual species of *Helianthemum*) has demonstrated the remarkable adaptability of these associations, which can result in the formation of (a) endomycorrhizae, characterized by undifferentiated coil-shaped or globular intracellular hyphae penetrating the plant cells [17,18,19,20]; (b) ectomycorrhizae, characterized by a Hartig net, but without a true sheath [18,21,22]; (c) ectendomycorrhizae, characterized by the presence of both intercellular Hartig net and intracellular hyphae penetrating the cortex cells [23,24]. Lately, it has been observed that in some instances, more than one of the above mycorrhizal types may be observed along the same root system of a single *Helianthemum* plant, a phenomenon that has been named “ectendomycorrhiza continuum” [23]. On the other hand, *Terfezia-Cistus* mycorrhizae seem to be consistently morphologically characterized as ectomycorrhizae with a well-developed Hartig net, however, the presence or absence of a true sheath is still subject of debate [5,16,25,26,27]. While most in vitro and ex vitro synthesis resulted in ectomycorrhizae with a well-developed Hartig net but without a true mantle [5], a more recent work provided evidence on the formation of ectomycorrhizae with a thin less-developed sheath using *C. salvifolius*, *C. albidus*, *C. incanus,* and three different *Terfezia* species [16]. In view of this new evidence, the main goal of the present work was to provide new insights on the association between *Terfezia* species and *Cistus* spp. by reporting on the mycorrhizae formed by four *Terfezia* species—namely *T. arenaria*; *T. extremadurensis*; *T. fanfani*, *T. pini*—along with *C. ladanifer* and *C. salviifolius*, two of the most widespread and common *Cistus* species in acidic soils. Furthermore, we aimed to assess which of the above *Terfezia-Cistus* combinations are the most compatible and open the possibility of mass production of *Terfezia* mycorrhized seedlings towards desert truffle cultivation in acid soils.

## 2. Materials and Methods

### 2.1. Fungal Material

Mature *Terfezia* ascocarps were harvested from different locations in the Alentejo (Southern Portugal), between February 2017 and April 2019. Ascocarps were identified to the species level (compared with the descriptions present in [28,29]) and fragments were isolated on LS medium [30] (in mg·L^−1^ 475 KNO_3_, 110 CaCl_2_·2H_2_O, 92.5 MgSO_4_·7H_2_O, 42.5 KH_2_PO_4_, 412 NH_4_NO_3_, 9.31 Na_2_EDTA, 6.96 FeSO_4_·7H_2_O, 4.22 MnSO_4_·4H_2_O, 2.15 ZnSO_4_·7H_2_O, 0.006 CuSO_4_·5H_2_O, 0.006 CoCl_2_·6H_2_O, 0.06 Na_2_MoO_4_·2H_2_O, 1.55 H_3_BO_3_, 0.21 KI; 0.025 thiamine hydrochloride; 0.125 nicotinic acid; 0.125 pyridoxine hydrochloride; 25 myo-inositol, 0.5 glycine, 0.5 6-benzylaminopurine (BAP), 0.5 indole-3-acetic acid (IAA)) with sucrose 10 g·L^−1^ and solidified with 10 g·L^−1^ of agar and pH 5.5. All isolates were incubated in the dark at 25 ± 2 °C for 90 days. Four isolates, with better growth rate, were selected for mycorrhization trials and molecular characterization: *T. arenaria* (Moris) Trappe strain 220, *T. extremadurensis* Muñoz-Mohedano, Ant. Rodr. & Bordallo strain 271, *T. fanfani* Matt. strain 235 and *T. pini* Bordallo, Ant. Rodr. and Muñoz-Mohedano strain 278.

Molecular characterization was carried out by sequencing fragments of the nuclear ribosomal DNA region of selected *Terfezia* mycelial cultures. DNA extractions were performed by a modified CTAB method [31]. The internal transcribed spacer (ITS) region of the rDNA, including the 5.8 S ribosomal gene, was amplified using the ITS5 and ITS4 primers [32]. Amplifications of ITS rDNA sequences were performed using a Mastercycler Gradient thermocycler (Eppendorf, Hamburg, Germany) with the following cycling parameters: an initial denaturalization step for 3 min at 95 °C, followed by 35 cycles consis-ting of: 30 s at 95 °C, 30 s at 95 °C (annealing temp.), 1 min at 72 °C, and a final extension at 72 °C for 10 min. All reagents were acquired from NZYTech, Lda, sequencing was done commercially (STAB VIDA, Lda.) and all sequence alignments were performed with online MAFFT version 7, using the E-INS-i strategy [33]. Molecular identification was carried out by comparing our sequences with the existing ones in the GenBank database.

### 2.2. Plant Material

Seeds from *C. salviifolius* L. and *C. ladanifer* L. plants growing in Herdade da Mitra, near Évora (Alentejo, Portugal) (38°32′ N; 8°01′ W; 220 m a.s.l.), were collected on November 2013 in a Montado area with natural shrub undercover dominated by *Cistus* spp. The area belongs to the Mediterranean pluviseasonal-oceanic bioclimate and is located in the low mesomediterranean bioclimatic belt. It has a dry to subhumid ombrotype of climate with a mean annual temperature ranging from 9.2 °C to 21.5 °C and a mean annual rainfall of 664.6 mm [34,35]. The collected *Cistus* seeds were dried at 23 °C in a Memmert forced ventilation oven (Model 600) and kept at room temperature in the dark until use. The seeds were surface sterilized by immersion in 70% ethanol for 2 min, followed by another immersion in a 50% bleach solution for 10–15 min. Afterward, seeds were washed three times in sterilized tap water. To break seed dormancy, seeds were heated at 150 °C in a bi-distilled water bath for 5 min and left to cool down until room temperature was reached. Seeds were then placed in Petri dishes, on top of the filter paper moistened with bi-distilled water, and kept in a growth chamber in the dark with 24 °C/21 °C (±1 °C) day/night temperature. After germination, *C. salviifolius* and *C. ladanifer* seedlings were routinely micropropagated using the *Cistus* rapid multiplication protocol described in [36].

### 2.3. Mycorrhizal Synthesis

Mycorrhizal synthesis was performed in polypropylene transparent microboxes (90 mm Ø and 120 mm in height) with filtered polypropylene covers. Each box containing 200 mL of dried vermiculite and 100 mL of LS liquid medium was autoclaved at 121 °C and 18 psi for 20 min. Mycelium pure cultures of *T. arenaria* (strain 220, Genbank MW356871), *T. extremadurensis* (strain 271, Genbank MW356873), *T. fanfani* (strain 235, Genbank MW356872), and *T. pini* (strain 278, Genbank MW356874) were used in the experiment.

**Figure 1:**
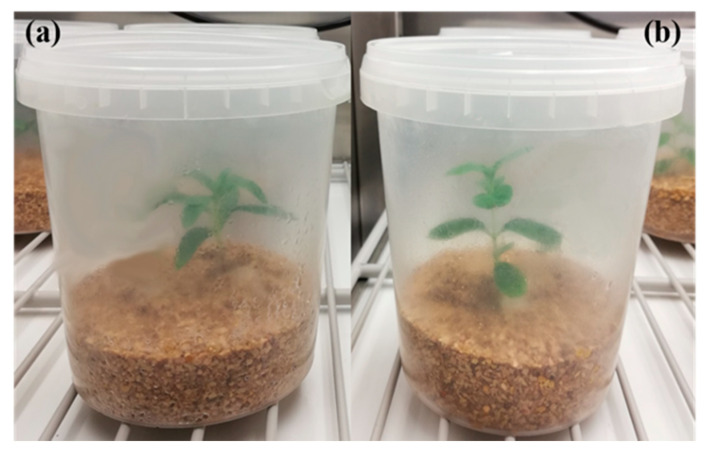
In vitro mycorrhizal system. (**a**) Detail of micropropagated *C. ladanifer* inoculated with *T. arenaria* mycelium; (**b**) detail of micropropagated *C. salviifolius* inoculated with *T. arenaria* mycelium.

After a week (to check for possible contaminants), sixteen boxes were inoculated with two plugs dissected from one of the selected *Terfezia* strains and incubated in the dark (25 ± 2 °C). The process was repeated for the four *Terfezia* species, totaling sixty-four boxes. Two weeks later, one rooted *Cistus* micropropagated plantlet (*C. salviifolius* or *C. ladanifer*) was introduced in each box, near the active growing mycelia, totaling eight replicates for each *Terfezia-Cistus* pair (Figure 1).

The boxes were then placed in the grow chamber at 24 °C/21 °C (± 1 °C) day/night temperature and 15 h light period, under cool white fluorescent light (36 µmol·m^−2^·s^−1^). After two months, the *Cistus* plantlets were carefully retrieved from the growing medium and their roots gently washed to free them from adhering particles. The whole root system of each *Cistus* plantlet was separated from the aerial part, kept in 50 mL centrifuge tubes filled with a glutaraldehyde solution (4%), and stored at 5 °C until further examination.

### 2.4. Mycorrhizal Morphotyping and Colonization Assessment

Each *Cistus* plantlet root system was washed over a 2 mm sieve and cut into segments of approximately 1 cm in length. Afterwards, the root segments were spread in two Petri dishes containing bi-distilled water and all root tips were observed under a stereo microscope (WILD M3) to determine the existent morphological types. Characterization of the mycorrhizal root tips followed [37,38]. Prior to the microscopic observation, all root fragments were cleared with a 10% KOH solution and stained with 0.1% trypan blue in lactophenol following the method developed by [39]. Microscopic examination of the root fragments and characterization of the mycorrhizal system under the light microscope was done, using a Leica DM750 microscope equipped with a digital camera (Leica ICC50W), according to the methodology described in [40]. The percentage of fungal root colonization was estimated based on the frequency of infection expressed by: FI (%) = 100 (N−N0)/N, where N is the total number of observed root fragments and N0 is the number of root fragments uninfected [41].

### 2.5. Statistical Analysis

Data normality was assessed using Kolmogorov–Smirnov tests. Levene’s tests were employed to assess the variance homocedasticity assumption. Arcsine data transformation was necessary to perform the two-way ANOVA. Mean differences in frequency of infection between plant hosts and different *Terfezia* isolates were tested through a two-way ANOVA followed by Tukey post hoc tests. All calculations were performed with IBM SPSS Statistics V 24 [42].

## 3. Results

**Figure 2:**
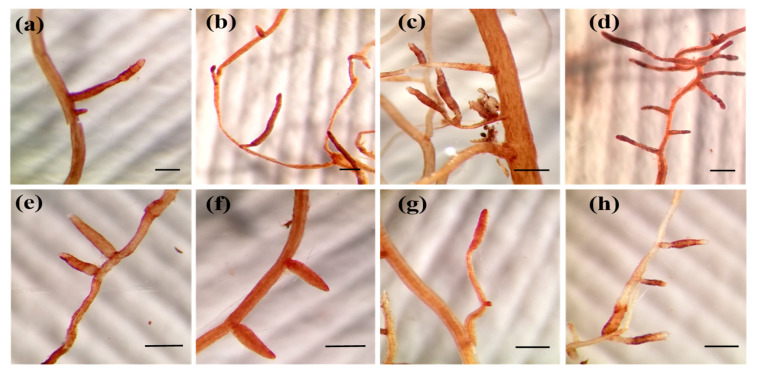
External characteristics of *Terfezia-Cistus* mycorrhizae, each scale bar measure 0.5 mm: (**a**) *T. arenaria* × *C. salviifolius* mycorrhizae, (**b**) *T. extremadurensis* × *C. salviifolius* mycorrhizae, (**c**) *T. fanfani* × *C. salviifolius* mycorrhizae, (**d**) *T. pini* × *C. salviifolius* mycorrhizae, (**e**) *T. arenaria* × *C. ladanifer* mycorrhizae, (**f**) *T. extremadurensis* × *C. ladanifer* mycorrhizae, (**g**) *T. fanfani* × *C. ladanifer* mycorrhizae, (**h**) *T. pini* × *C. ladanifer* mycorrhizae.

All *Cistus* plantlets, irrespective of the host plant–fungal species combination, formed mycorrhizal associations with all the *Terfezia* isolates after two months using the mycorrhizal system described in material and methods section. Concerning the macroscopic morphological characterization of the mycorrhizal root tips, a single morphotype was produced by every host plant–fungal species combination (Figure 2). Under the stereomicroscope, mycorrhizae were unbranched, unramified ends were straight to bent, more or less inflated, sometimes with a more enlarged apex (club shaped). Surface of unramified ends was smooth, color varied from brownish-yellow to rusty-brown ochre, with slightly darker tones on aged mycorrhizae. Emanating hyphae were infrequent, white, and shiny. No rhizomorphs were observed.

**Figure 3:**
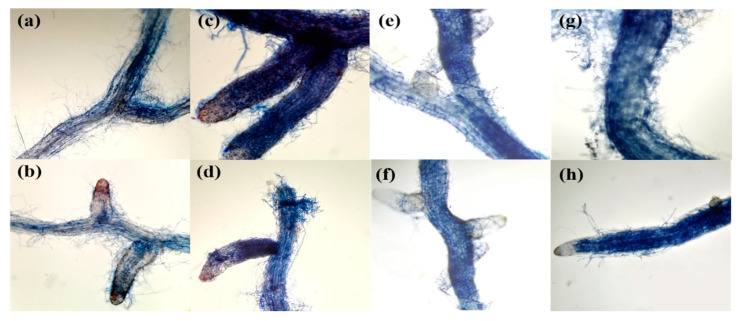
Light microphotographs of *Terfezia-Cistus* mycorrhizal roots, (400×): (**a**) *T. arenaria × C. salviifolius* root showing rudimentary sheath, (**b**) *T. arenaria* × *C. ladanifer* root showing rudimentary sheath, (**c**) *T. extremadurensis* × *C. salviifolius* root surrounded by a well-developed sheath, (**d**) *T. extremadurensis* × *C. ladanifer* root showing well-developed sheath, (**e**) *T. fanfani* × C. *salviifolius* root showing a less-developed sheath, (**f**) *T. fanfani* × *C. ladanifer* root showing a less-developed sheath, (**g**) *T. pini* × *C. salviifolius* root surrounded by a diffuse sheath, (**h**) *T. pini* × *C. ladanifer* root showing a well-developed sheath.

**Figure 4:**
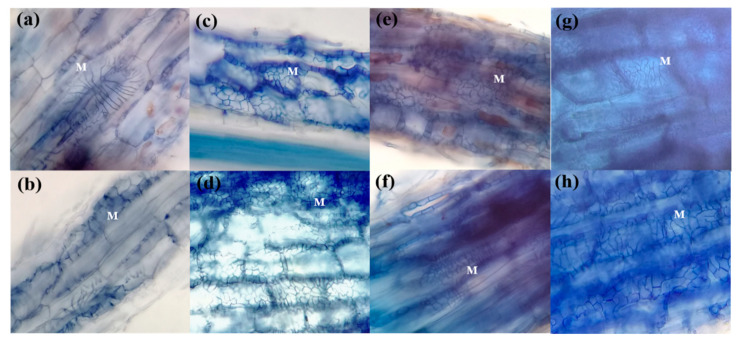
Light microphotographs of *Terfezia-Cistus* mycorrhizal roots, (400×): (**a**) detail of *T. arenaria* × *C. salviifolius* ectomycorrhizae mantle structure (M), (**b**) detail of *T. arenaria* × *C. ladanifer* ectomycorrhizae mantle structure (M), (**c**) detail of *T. extremadurensis* × *C. salviifolius* ectomycorrhizae mantle structure (M), (**d**) detail of *T. extremadurensis* × *C. ladanifer* ectomycorrhizae mantle structure (M), (**e**) detail of *T. fanfani* × *C. salviifolius* ectomycorrhizae mantle structure (M), (**f**) detail of *T. fanfani* × *C. ladanifer* ectomycorrhizae mantle structure (M), (**g**) detail of *T. pini* × *C. salviifolius* ectomycorrhizae mantle structure (M), (**h**) detail of *T. pini* × *C. ladanifer* ectomycorrhizae mantle structure (M).

**Figure 5:**
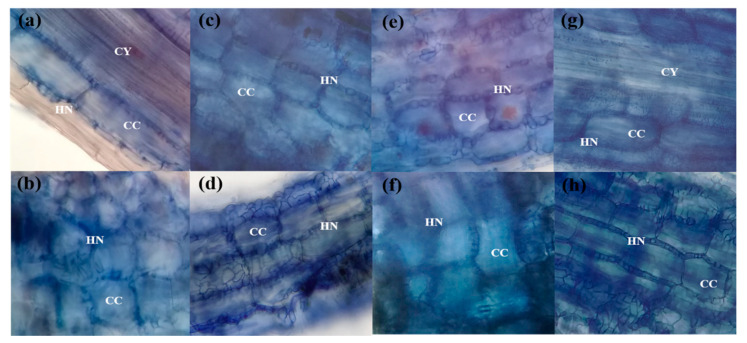
Light microphotographs of *Terfezia-Cistus* mycorrhizal roots, (400×): (**a**) *T. arenaria* x *C. salviifolius* ectomycorrhizae showing Hartig net (HN) restricted to cortical cells (CC), (CY) central cylinder; (**b**) *T. arenaria* x *C. ladanifer* ectomycorrhizae showing well-developed Hartig net (HN) surrounding cortical cells (CC); (**c**) *T. extremadurensis* x *C. salviifolius* ectomycorrhizae showing intercellular hyphae pearl structure (Hartig net (HN)) between cortical cells (CC); (**d**) *T. extremadurensis* x *C. ladanifer* ectomycorrhizae showing well-developed Hartig net (HN) between cortical cells (CC); (**e**) *T. fanfani* x *C. salviifolius* ectomycorrhizae showing the characteristic pearl structure of the Hartig net (HN) surrounding root cortical cells (CC); (**f**) *T. fanfani* x *C. ladanifer* ectomycorrhizae with well-developed Hartig net (HN) between cortical cells (CC); (**g**) *T. pini* x *C. salviifolius* ectomycorrhizae showing well-developed Hartig net (HN) surrounding cortical cells (CC), (CY) central cylinder; (**h**) *T. pini* x *C. ladanifer* ectomycorrhizae showing a widespread Hartig net (HN) surrounding cortical cells (CC).

Additional microscopic examination of the root fragments revealed that all four *Terfezia* species (i.e., *T. arenaria*, *T. fanfani*, *T. extremadurensis,* and *T. pini*) formed ectomycorrhizae with a well-developed Hartig net but with varying degrees of mantle development (Figure 3, Figure 4 and Figure 5), both with *C. salviifolius* and *C. ladanifer*.

Overall, the mycorrhizae formed between the different *Terfezia-Cistus* associations displayed similar microscopic characteristics irrespective of host plant–fungal species association. A general description of the anatomic features of these associations is provided below.

The outer mantle structure with a densely plectenchymatous to nearly pseudoparenchymatous structure was composed of colorless angular cells, which were more marked in *T. extremadurensis* and *T. pini*, but also noticeable in *T. fanfani*. The inner mantle plectenchymatous was characterized by colorless hyphae forming a coarse net of irregularly shaped hyphae, tightly glued together, which sometimes begin as small star-like arrangements, as in the case of *T. arenaria* (Figure 4a,b).

The Hartig net is usually composed of a single row of hyphae that protrudes deeply towards the endodermis, enveloping completely one to three rows of cortical cells, but never touching the endodermis or the central cylinder. Hyphal segments around cortical cells are initially of constant width but later forming a beaded or pearl-like structure (Figure 5c,e).

**Figure 6:**
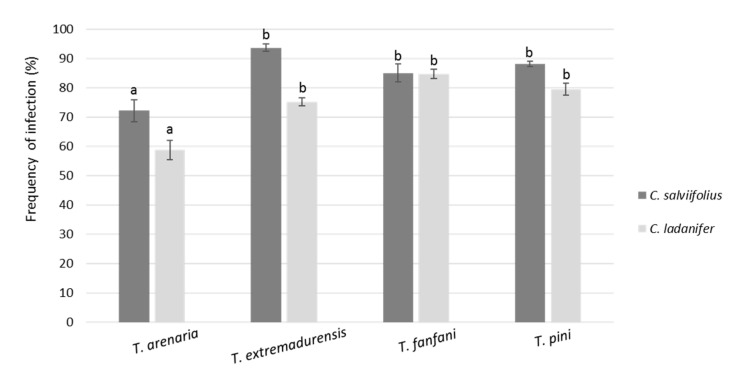
Frequency of infection by the four *Terfezia* isolates (means ± SE, n = 8) on root tips of *C. salviifolius* and *C. ladanifer*. Different letters over the bars represent significant differences between the means among different *Terfezia-Cistus* combinations using two-way ANOVA followed by Tukey test (*p* < 0.05). Two-way ANOVA results for *Terfezia* (T) frequency of infection in *Cistus* (C), T: F = 27.42 ***, C: F = 41.59 ***, T X C: F = 6.36 ***. Significance level *** *p* < 0.001.

The two-way ANOVA showed that mean frequencies of infection were significantly influenced by the *Terfezia* species, the *Cistus* species, and their interaction (Figure 6). As such, regarding the mycosimbiont, *T. arenaria* showed significantly lower mean frequencies of infection then the other three *Terfezia* species. Concerning plant host, *C. salviifolius* revealed higher frequencies of infection with *T. arenaria*, *T. extremadurensis,* and *T. pini*, compared to *C. ladanifer*. However, no clear differences in the mean frequencies of infection were found between plant hosts for *T. fanfani*.

## 4. Discussion

The present work has brought forward compelling evidence on the ability of *T. arenaria*, *T. extremadurensis*, *T. fanfani,* and *T. pini* to engage in mycorrhizal association under in vitro culture conditions, with two of the most widespread *Cistus* species (i.e., *C. salviifolius* and *C. ladanifer*). Furthermore, it provides for the first time a comprehensive macro and microscopic descriptions of the mycorrhizae formed between *T. arenaria*, *T. extremadurensis,* and *T. pini* on the above mentioned *Cistus* species.

One interesting question that needed answering was the presence or absence of a true sheath in these mycorrhizal associations. We can now ascertain that all four *Terfezia* species analyzed (i.e., *T. arenaria, T. fanfani, T. extremadurensis,* and *T. pini*) do form ectomycorrhizae with a true sheath, and therefore agree with the work of [16]. However, differences in mantle development were observed between the mycorrhizae formed by the four mycosymbionts, with *T. arenaria* colonized roots showing only a sparsely rudimentary sheath, whereas on the other end, *T. extremadurensis* colonized roots where surrounded by a profuse well-developed sheath, under the same experimental conditions. Although these differences might represent true differences on the morphological characters of those particular associations, another possible explanation is that the observed differences might just be a reflection of the flexibility of each *Terfezia* species to colonize different plant hosts. In other words, although capable of entering into the mycorrhizal association, the time or the conditions required to form fully developed mycorrhizae may differ between *Terfezia* species. For instance, this might indicate that *T. arenaria* have a narrower putative host range than the remaining *Terfezia* in the analysis.

In summary, given that modern truffle cultivation is largely based on mass production of adequately colonized plants raised under controlled conditions [43], our results are encouraging since all eight *Terfezia-Cistus* combinations expressed high rates of mycorrhization (comparable to those obtained in previous works) [16,27,44]. Nevertheless, our experimental data seem to indicate that *C. salviifolius* is a better option than *C. ladanifer* as potential host for the production of *T. arenaria* inoculated plants, and its subsequent application on desert truffle cultivation. These results are encouraging since *T. arenaria* sporocarps are one of the most prized and traded desert truffle worldwide.

Yet, the choice of the fungal partner in truffle cultivation depends on various factors (e.g., sporocarp size, edibility, plantation purpose, interactions with other organisms, etc.). Regarding the tested mycosymbionts, *T. fanfani* seems to be the best option towards desert truffle cultivation in acid soils, mostly due to its ascocarp size, gastronomic value, and its capacity to infect both hosts.

Nevertheless, the introduction of *T. extremadurensis* and *T. pini* inoculated *Cistus* seedlings may also be interesting alternatives for reforestation programs and/or to prevent soil erosion after intense disturbances.

Though these results are promising for trufficulture, further studies are still needed to ascertain if the considered *Terfezia-Cistus* combinations will result in the formation of sporocarps under field conditions. In that regard, we already obtained some preliminary results in an experimental plot where we confirmed the persistence of *Cistus* inoculated mycorrhizae and sporocarp production [45] two years after plant installation on the plot.

## Data Availability

Not applicable.
